# Hyperoxia in intensive care, emergency, and peri-operative medicine: Dr. Jekyll or Mr. Hyde? A 2015 update

**DOI:** 10.1186/s13613-015-0084-6

**Published:** 2015-11-19

**Authors:** Sebastian Hafner, François Beloncle, Andreas Koch, Peter Radermacher, Pierre Asfar

**Affiliations:** Institut für Anästhesiologische Pathophysiologie und Verfahrensentwicklung, Universitätsklinikum Ulm, Helmholtzstrasse 8-1, 89081 Ulm, Germany; Klinik für Anästhesiologie, Universitätsklinikum Ulm, Albert-Einstein-Allee 23, 89081 Ulm, Germany; Département de Réanimation Médicale et de Médecine Hyperbare, Centre Hospitalier Universitaire, 4 rue Larrey, Cedex 9, 49933 Angers, France; Laboratoire de Biologie Neurovasculaire et Mitochondriale Intégrée, CNRS UMR 6214-INSERM U1083, Université Angers, PRES L’UNAM, Nantes, France; Sektion Maritime Medizin, Institut für Experimentelle Medizin, Christian-Albrechts-Universität, 24118 Kiel, Germany; Schifffahrtmedizinisches Institut der Marine, 24119 Kronshagen, Germany

## Abstract

This review summarizes the (patho)-physiological effects of ventilation with high FiO_2_ (0.8–1.0), with a special focus on the most recent clinical evidence on its use for the management of circulatory shock and during medical emergencies. Hyperoxia is a cornerstone of the acute management of circulatory shock, a concept which is based on compelling experimental evidence that compensating the imbalance between O_2_ supply and requirements (i.e., the oxygen dept) is crucial for survival, at least after trauma. On the other hand, “oxygen toxicity” due to the increased formation of reactive oxygen species limits its use, because it may cause serious deleterious side effects, especially in conditions of ischemia/reperfusion. While these effects are particularly pronounced during long-term administration, i.e., beyond 12–24 h, several retrospective studies suggest that even hyperoxemia of shorter duration is also associated with increased mortality and morbidity. In fact, albeit the clinical evidence from prospective studies is surprisingly scarce, a recent meta-analysis suggests that hyperoxia is associated with increased mortality at least in patients after cardiac arrest, stroke, and traumatic brain injury. Most of these data, however, originate from heterogenous, observational studies with inconsistent results, and therefore, there is a need for the results from the large scale, randomized, controlled clinical trials on the use of hyperoxia, which can be anticipated within the next 2–3 years. Consequently, until then, “conservative” O_2_ therapy, i.e., targeting an arterial hemoglobin O_2_ saturation of 88–95 % as suggested by the guidelines of the ARDS Network and the Surviving Sepsis Campaign, represents the treatment of choice to avoid exposure to both hypoxemia and excess hyperoxemia.

## Background

The “double-edged sword” character of molecular oxygen (O_2_) is well established and has been a matter of debate since its discovery at the end of the eighteenth century. On the one hand, O_2_ plays a crucial role during adenosine triphosphate (ATP) synthesis [1]. On the other hand, its chemical characteristics lead to strong oxidizing properties, capable of damaging any biological molecule [1], and thereby defining the paradigm of oxygen toxicity. This phenomenon is due to the formation of reactive oxygen species (ROS), its magnitude being directly correlated to the level of the O_2_ partial pressure [2, 3]. Moreover, during mitochondrial respiration, 1–3 % of O_2_ consumption leads to ROS formation [3]. Like O_2_, ROS also exert Janus-headed properties: while being of importance for host defense and signaling cascades, their toxic effects are well known [4].

**Fig. 1:**
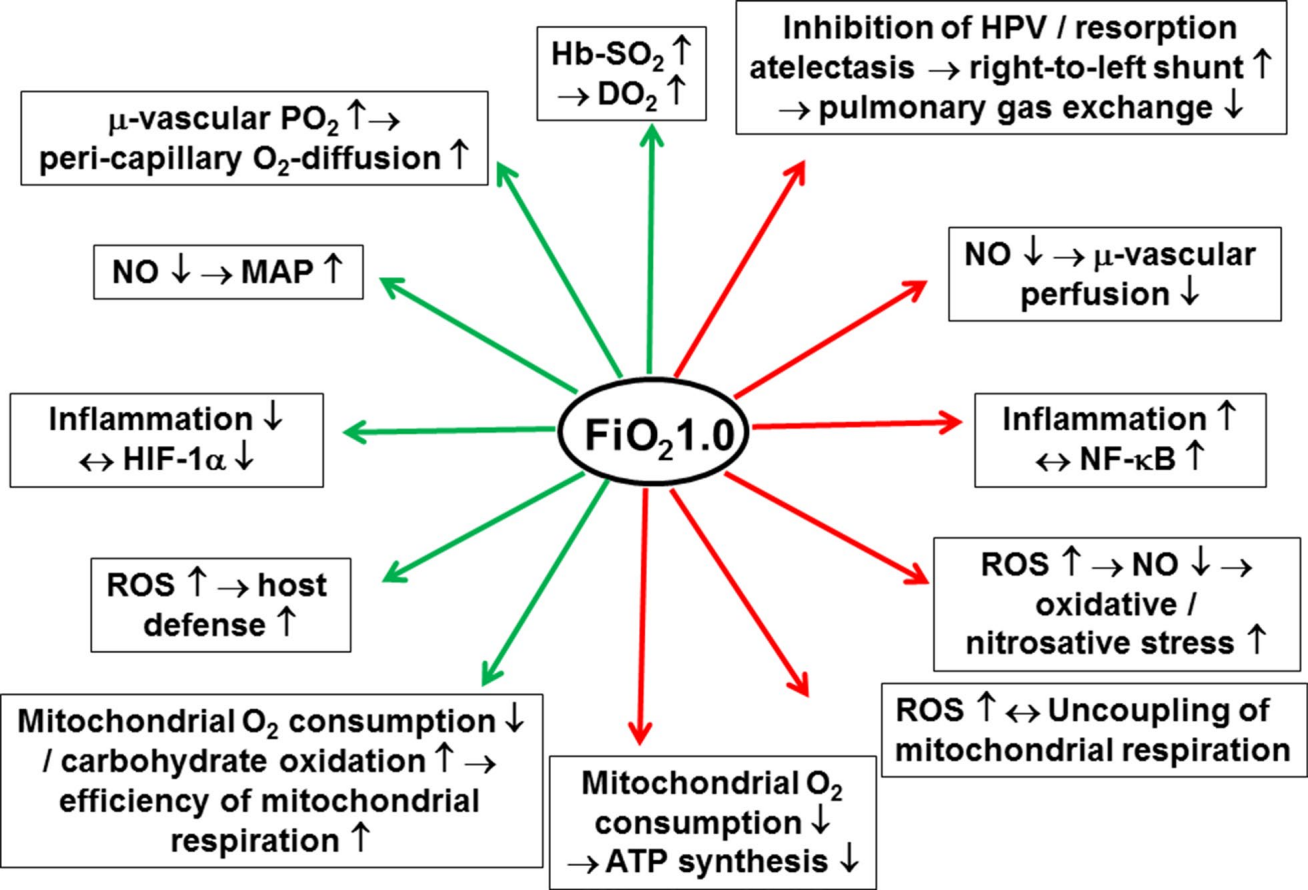
Beneficial (*green arrows*) and deleterious (*red arrows*) effects of hyperoxia, i.e., breathing pure oxygen, during circulatory shock and/or in medical emergencies. *FiO*_*2*_ fraction of inspired oxygen, *PO*_*2*_ oxygen partial pressure, *µ* micro, *Hb* haemoglobin, *SO*_*2*_ oxygen saturation, *DO*_*2*_ systemic oxygen transport, *HPV* hypoxic pulmonary vasoconstriction, *MAP* mean arterial pressure, *SVR* systemic vascular resistance, *NO*: nitric oxide, *HIF*-*1α*: hypoxia-inducible factor 1 alpha, *NF*-*κB* nuclear transcription factor kappaB, *ROS* reactive oxygen species, *ATP* adenosine triphosphate; adapted from Asfar et al. [16] with kind permission from Springer Science and Business Media

Circulatory shock is defined as “…an imbalance between O_2_ supply and requirements*…*” [5], and consequently, a logical therapeutic strategy is to increase the inspired O_2_ concentration (FiO_2_). The recommendation that the administration of oxygen should be started immediately to increase O_2_ delivery [6] has been known for a long time as a part of the “V” (“ventilate”) component of the “VIP” (“ventilate–infuse–pump”) rule for shock resuscitation [6]. Due to this, supplemental O_2_ was an integral part of all resuscitation protocols of the recently published Protocolized Care for Early Septic Shock (ProCESS) trial [7]. However, literature data concerning the high-dose administration of O_2_ are still highly controversial [8–14]. Moreover, hyperoxia (i.e., an increased FiO_2_) must be distinguished from hyperoxemia (i.e. increased arterial O_2_ partial pressure): in patients with severe Acute Respiratory Distress Syndrome (ARDS), hyperoxia may be mandatory to avoid hypoxemia with the least mechanical ventilation-induced hemodynamic compromise and/or ventilator-induced damage to the lung possible. This is nicely demonstrated by the results of the ARDSNetwork trial on low tidal volume ventilation: the FiO_2_ was significantly higher in the group with lower tidal volumes that ultimately had improved survival [15]. Figure 1 [16] summarizes the key pro and con arguments concerning the use of O_2_ therapy during shock states. Data from prospective, controlled, randomized trials on the use of therapeutic hyperoxia, however, are surprisingly scarce. Consequently, given the possible deleterious side effects of hyperoxia, the current guidelines of the ARDSNetwork and the Surviving Sepsis Campaign recommend using an FiO_2_ that allows achieving an arterial hemoglobin O_2_ saturation of 88–95 % at airway plateau pressures and PEEP levels of <30 and 5–20 cmH_2_O, respectively [15, 17]. The present review will, therefore, discuss the role of ventilation with high FiO_2_ (0.8–1.0) during circulatory shock, during medical emergencies and in the peri-operative period; the first part will briefly summarize the (patho)-physiological effects of hyperoxia, the second part will review its use in the context of important pathological entities, with a particular focus on the most recent clinical evidence.

## (Patho)physiology of hyperoxia: pulmonary, vascular, metabolic, and cerebral effects

### Blood O_2_ content

According to textbook physiology, increasing the FiO_2_ from 0.21 (i.e., air) to 1.0 (i.e., 100 % O_2_) will moderately affect total blood O_2_ content under conditions of normal cardiopulmonary function: at normal pH and temperature, arterial PO_2_ levels of 90–100 mmHg lead to hemoglobin O_2_ saturations close to 100 % due to the sigmoid shape of the hemoglobin-O_2_-dissociation curve. Therefore, pure O_2_ breathing will only raise the amount of physically dissolved O_2_, the maximum effect being a five-fold increase, while hardly modifying the amount of O_2_ bound to hemoglobin. It is self-evident from the afore-mentioned estimate that the effect of pure O_2_ breathing on total blood O_2_ content will be the more important the lower the hemoglobin concentration. Therefore, ventilation with 100 % O_2_ was particularly protective in various models comprising critical hemodilution (reviewed in Calzia et al. [12]): the most impressive evidence in this context are the data reported in the “Live without blood” experiment as early as in 1960 [18]: in pigs subjected to hemodilution to a hematocrit <1–2 % (!), mechanical ventilation with pure O_2_ allowed preventing the otherwise marked ECG signs of myocardial ischemia, and no sequelae were observed after blood re-transfusion and return to air breathing. Strikingly, however, despite its frequent routine use, so far there are no clinical data on the role of mechanical ventilation with FiO_2_ = 1.0 during the management hemorrhagic shock, most likely due to ethical constraints. The available pre-clinical data are equivocal: deleterious and beneficial effects as well as no therapeutic efficacy at all were reported, depending on the species used, the severity of shock, and the concomitant use of therapeutic hypothermia [19–25].

No matter the definitive role of pure O_2_ breathing during situations of critical reductions in blood O_2_ transport capacity due to blood loss and/or hemodilution, pre-oxygenation, i.e., administration of 100 % O_2_ prior to induction of anesthesia and/or airway management, is well established to markedly increase the margin of safety: the “safe time of apnea” (i.e., the time until transcutaneous O_2_ saturation fell below 90 %) was doubled, when the FiO_2_ was increased from 60 to 100 % [26]. It must be noted, however, that even short-term pure O_2_ ventilation under these conditions may be associated with formation of atelectasis (see below). In healthy, non-obese patients with American Society of Anesthesiologists physical status I or II, this atelectasis formation was prevented by using an FiO_2_ of 0.8, but the safe time of apnea was significantly shorter [26]. Unfortunately, there is no ideal FiO_2_, which allows achieving a maximum “safe time of apnea” with the least formation of atelectasis [27, 28]: the degree of the latter depends on the patients’ age [29], body mass index [30], and underlying chronic pulmonary co-morbidity [31].

Interestingly, this concept of a prolonged margin of safety seems to be valid in coronary artery disease for O_2_ breathing as a preventive, pre-treatment measure: breathing 15 L/min O_2_ prevented the recurrence of pacing- [32] and prolonged the time until occurrence of exercise-induced angina [33].

### Pulmonary effects

Pure O_2_ breathing impairs pulmonary gas exchange as a result of inhibition of hypoxic pulmonary vasoconstriction induced by the rise in alveolar and mixed-venous PO_2_ [34, 35]. Moreover, as already mentioned-above, within a few minutes, e.g., after only 5 min of apnea and oxygenation during induction of anesthesia [26], pure O_2_ breathing causes formation of atelectasis with increased intrapulmonary right-to-left shunt. This “adsorption atelectasis” [36, 37] is due to instability of lung regions that are still open but poorly ventilated in relation to perfusion, so-called low ventilation/perfusion-ratio (V_A_/Q) regions [38], when the inert carrier gas N_2_ is washed out. In healthy volunteers, breathing 100 % O_2_ over approx. 25 min under normobaric conditions doubled intra-pulmonary right-to-left shunt, while breathing air at equal inspiratory O_2_ partial pressure, i.e., in a hyperbaric chamber pressurized to 4.9 atmospheres of ambient pressure, did not affect gas exchange [12]. During induction of anesthesia, this atelectasis formation was prevented at least in part by using CPAP breathing and subsequent face mask ventilation with a PEEP of 6–10 cmH_2_O [39, 40]. In mechanically ventilated patients with acute lung injury, the degree of hyperoxia-induced adsorption atelectasis could at least be attenuated by using higher PEEP levels: increasing PEEP from 5 to 14 cm H_2_O completely blunted the fall of the PaO_2_/FiO_2_ ratio from 200 to 150 mm Hg induced by increasing the FiO_2_ from 0.6 to 1.0 [37].

Acute hyperoxia-induced impairment of gas exchange must be discriminated from pulmonary O_2_ toxicity [41, 42], the so-called *Lorrain*-*Smith* effect [43] first described by Lavoisier in 1783 [44]. Pulmonary O_2_ toxicity may present as severe pulmonary inflammation, ultimately leading to hemorrhagic pulmonary edema, and is referred to excess ROS and reactive nitrogen species (RNS) formation [45, 46]. However, despite the abundant evidence on hyperoxia-induced acute lung injury from studies in experimental animal (for reviews see [45, 47]), so far no biomarkers have been identified in humans that would allow evaluating the degree of ROS and/or RNS formation, and, moreover, thereby avoiding pulmonary O_2_ toxicity. Consequently, albeit intuitively being a logical therapeutic approach, there are no large scale data on the prevention of pulmonary O_2_ toxicity by ROS scavengers in humans, similar to the equivocal role of antioxidants in critically ill patients in general [48, 49]. In healthy experimental animals, pulmonary O_2_ toxicity is a result of either long-term exposure and/or injurious ventilator settings leading to ventilator-induced lung injury (VILI) (for examples, see [50, 51]). In contrast, lung-protective ventilation using low tidal volumes with higher PEEP levels and/or titrated to the thoraco-pulmonary compliance curve [52] over shorter periods had no deleterious effect at all [53], and this was also true during 24 h of lung-protective ventilation at FiO_2_ 1.0 in in large animals [25, 54]. In humans, the duration of pure O_2_ breathing needed to provoke pulmonary O_2_ toxicity is unknown [55]: various studies reported that exposure period of 6–25 h were associated with clinical and histological signs of tracheitis and/or alveolitis [45, 56, 57], whereas other authors suggested that “*…*direct oxygen toxicity only plays a negligible role in regards to perioperative administration..” [58] and that breathing an FiO_2_ of 0.96–1.0 for 48 h does not produce symptoms of toxicity in most men [45, 59]. The only data available from mechanically ventilated ICU patients originate from mechanical ventilation with FiO_2_ >0.85–0.9 for >10 days [60, 61], and FiO_2_ = 1.0 over 14 h to 30 days [62, 63], respectively. Unfortunately, the studies do not report ventilator settings, but, given the publication years (1967–1972), it is unlikely that low tidal volumes and high PEEP levels according to current guidelines were used. Two more recent studies yielded equivocal results: observational data from patients mechanically ventilated for >48 h with “excessive inspired O_2_” (defined as an “FiO_2_ >0.5 while maintaining SO_2_ >92 %” observed in 155 out of 210 patients during a 12-month observation period) showed significantly lower PaO_2_/FiO_2_ ratio and higher mean airway pressures at 48 h [64]. In contrast, retrospective analyses of patients after cardiac arrest showed that higher quartiles of the “area under curve of FiO_2_” were not associated with any effect on gas exchange or lung mechanics during the first 24 h of mechanical ventilation [65]. Nevertheless, in this study the highest quartile of the “area under curve of FiO_2_” coincided with decreased survival to hospital discharge and worse neurological outcomes. Hence, no threshold value for the duration of hyperoxia exposure leading to pulmonary O_2_ toxicity is known in mechanically ventilated patients. Most likely, defining such a threshold is per se impossible: it is well known from hyperbaric (patho)physiology that intermittent exposure to hyperoxia with interspersed short periods of air breathing markedly attenuates pulmonary O_2_ toxicity when compared to an equally long, but continuous exposure [66]. The problem of defining a threshold value for the initiation of pulmonary O_2_ toxicity was highlighted during the discussion of “Oxygen” during the 50th Respiratory Care Journal Conference held April 13–14, 2012, in San Francisco, CA: “…oxygen toxicity is like Bigfoot: everybody’s heard about it, but nobody’s ever seen it…” [45].

### Vascular effects

Hyperoxia decreases cardiac output, on the one hand due to a fall in heart rate caused by increased parasympathetic tone [67], on the other hand due to a rise in systemic vascular resistance [68–70]. The latter may result from decreased ATP release from red blood cells [71] and/or reduced NO bioavailability. Stamler et al. elegantly demonstrated that the hyperoxia-induced increase in tissue and, consequently, venous PO_2_ levels blocks the release of NO from cystein-binding in the hemoglobin-molecule (*S*-nitrosothiol) [72]. In addition, increased ROS formation contributes to hyperoxia-induced vasoconstriction: administration of vitamin C (200 mg intra-arterial [69] and 3 g intra-venous [73], respectively), restored forearm [69] and coronary vascular resistance [73]. While varying among the different vascular regions, the degree of the hyperoxia-induced vasoconstriction is particularly pronounced in the cerebral and coronary circulation. Therefore, it was argued that this hyperoxia-related vasoconstriction may impede tissue O_2_ delivery in patients with sepsis [74] or cardiovascular disease [75], but it is still a matter of debate whether the hyperoxia-induced vasoconstriction is beneficial or deleterious: in fact, 30 min of pure O_2_ breathing impaired the sublingual microcirculatory perfusion by decreasing the number and density of perfused vessels, while it even increased perfusion heterogeneity [76]. It must be noted, however, that most of the studies available in the literature on hyperoxia-induced systemic or regional vasoconstriction were performed in healthy volunteers or at least under stable hemodynamic conditions, i.e., without imbalance between O_2_ supply and demand, or, during circulatory shock. In addition, any hyperoxia-related increase in vasomotor tone could possibly allow reducing vasopressor demands required to counteract shock-induced hypotension. Finally, experimental data suggest that pure O_2_ ventilation may redistribute cardiac output in favor of the kidney and the hepato-splanchnic system and thereby improve visceral organ function [25, 54, 77]. Yet, scarce data are only available on the effects of ventilation with FiO_2_ = 1.0 on systemic or regional hemodynamics and organ function in patients with circulatory shock. Only one prospective pilot study, including 83 patients admitted to the emergency department with two or more systemic inflammatory response syndrome (SIRS) criteria and a suspected infection, i.e., sepsis, reported no association between in-hospital mortality and hyperoxia (FiO_2_ between 0.4 and 0.8) [78], but only three patients with septic shock were included in total. Therefore, the results of the prospective, randomized, controlled HYPER2S (NCT01722422) trial (see Table 1) will certainly help to answer this question.

**Table 1:** Clinical trials on the effects of hyperoxia in intensive care and emergency medicine

Study acronym	Trial no.	Patient condition	Intervention	Primary outcome measures	Planned enrolment
OXYGEN-ICU	NCT01319643	ICU treatment for 3 days	FiO_2_ titrated to SpO_2_ 94–98/PaO_2_ 70–100 mmHg vs. SpO_2_ >97 %/PaO_2_ 100–150 mmHg	Mortality day 30	Terminated at n = 434 (slow recruitment)
HYPER2S	NCT01722422	Septic shock	FiO_2_ titrated to SpO_2_ 88–95 % vs. FiO_2_ = 1.0 over the first 24 h	Mortality day 28	Terminated at n = 442
AVOID	NCT01272713	Acute myocardial infarction	Air (unless SpO_2_ <94 %) vs. 8 L/min O_2_ during pre-hospital phase, thereafter according to hospital protocol	Infarct size, time course of CK-MB and cTnI	Completed at n = 638
DETO2X-AMI	NCT01787110	Acute coronary syndrome	Air (unless SpO_2_ <90 %) vs. 6 L/min O_2_ over 6–12 h	Mortality at 1 year	6600
BRAINOXY	NCT01201291	TBI, GCS ≤8	FiO_2_ 0.4 vs. 0.7	GOS/GOSE at 6 months	n un-specified; terminated (slow recruitment)
SO_2_S	ISRCTN52416964	Stroke, ICH	Air vs. 2 (SpO_2_ >93 %)/3 L/min overnight vs. 2 (SpO_2_ >93 %)/3 L/min continuously until day 3	Modified Rankin scale at day 90	Completed at n = 8003
REOX	NCT01881243	Cardiac arrest	Observational study; association between hyperoxia and outcome	Blood isofuranes/-prostanes	133

*ICU* intensive care unit, *FiO*_*2*_ fraction of inspired O_2_ concentration, *SpO*_*2*_ transcutaneous hemoglobin O_2_ saturation, *PaO*_*2*_ arterial O_2_ partial pressure, *CK-MB* myocardial creatine kinase, *cTnI* cardiac troponin I, *TBI* traumatic brain injury, *GCS* Glasgow Coma Score, *GOS* Glasgow Outcome Score, *GOSE* Extended Glasgow Outcome Score

### Metabolic effects

No matter the definitive effect of hyperoxia on vascular tone during circulatory shock, any conclusion on the role of hyperoxia-induced vasoconstriction must be considered in the context of the effects of hyperoxia on metabolic activity. Clearly, in vitro long-term (≥24 h) exposure to hyperoxia was associated with impaired mitochondrial respiratory capacity as a result of partial inhibition of NADH and succinate dehydrogenase, i.e., complex I and II [79, 80], whereas cytochrome c oxidase (complex IV) remained unaffected [79]. Pure O_2_ breathing also decreased whole body O_2_ uptake in healthy volunteers [76, 81] as well as in critically ill patients [70, 82], and myocardial O_2_ consumption in patients with coronary artery disease [83]. Nevertheless, this reduced O_2_ uptake more likely mirrored decreased O_2_ demand rather than impaired O_2_ utilization: There was no deleterious effect on any marker of systemic energy balance [70, 82], and myocardial lactate extraction was even enhanced [83]. Moreover, studies in experimental animals [25, 54] and healthy volunteers [81] showed that hyperoxia increased the respiratory quotient to values close to 1.0, in other words suggesting that hyperoxia shifted energy metabolism to preferential utilization of carbohydrates [25, 54], which is well established to increase the yield of the mitochondrial respiratory chain [84], i.e., the molar ratio of O_2_ consumption and ATP formation [85]. Similar to the situation during exercise in highlanders [86], this effect might assume particular importance under conditions of limited tissue O_2_ supply, e.g., hemorrhagic and/or cardiogenic shock.

### Cerebral effects

**Fig. 2:**
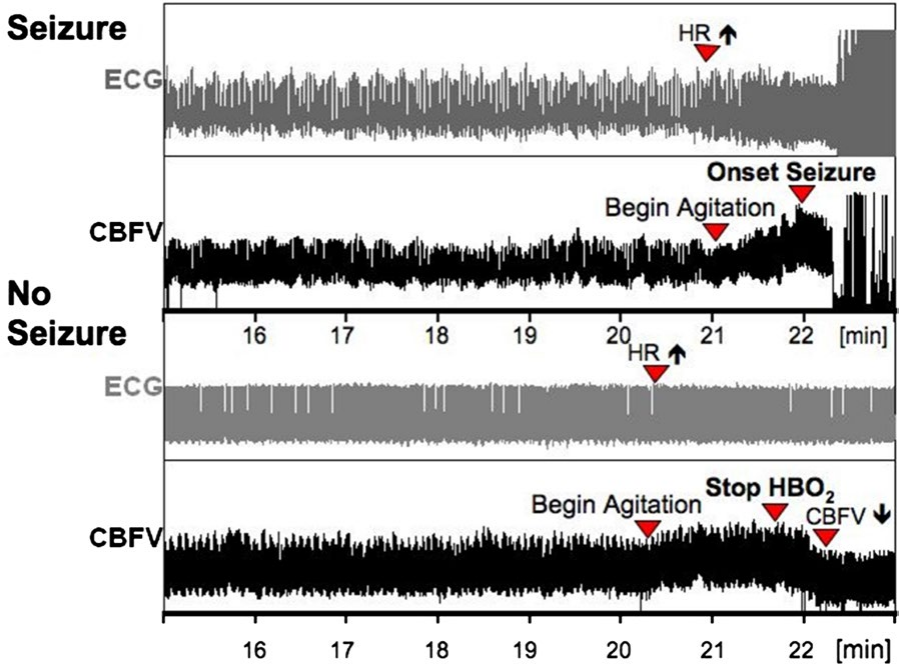
Original recordings of ECG and cerebral blood flow velocity (CBFV) in two volunteers undergoing an HBO exposure-test with pure O_2_ breathing at three atmospheres of ambient pressure. In the* upper panel*, HBO-induced seizures were preceded by tachycardia, agitation, and a subsequent marked increase in CBFV. The* lower panel* shows a volunteer, in whom seizures could be prevented by removing the O_2_ face mask; CBFV consecutively fell to lower levels comparable to those during the asymptomatic period

In addition to the above-mentioned pulmonary toxicity, pure O_2_ breathing may also have toxic effects on the central nervous system, the so-called *Paul*-*Bert* effect [87], the most dramatic manifestation being generalized tonic–clonic (grand mal) seizures [11]. This central nervous toxicity, however, requires pure O_2_ breathing under supra-atmospheric pressures, i.e., during diving and/or in a hyperbaric chamber. Hence, only critically ill patients treated with hyperbaric oxygenation (HBO: pure O_2_ breathing at supra-atmospheric pressures; e.g., for decompression injury (DCI), gas embolism, carbon monoxide (CO) poisoning, and gas gangrene or necrotizing fasciitis) will present with central nervous O_2_ toxicity-induced convulsions, which occur within approx. 20–30 min of pure O_2_ exposure at ambient pressures of three atmospheres. Interestingly, in contrast to the cerebral vasoconstriction normally observed during pure O_2_ breathing, symptoms are preceded by a paradoxical increase in cerebral blood flow velocity [88] (Fig. 2), which is referred to peroxynitrite (ONOO^−^) formation resulting from the reaction of NO with the superoxide radical (O_2_^−^) [89], and thereby causing a dysregulation of the endogenous NO availability [46, 90].

## Clinical application of hyperoxia

### CO intoxication, gas embolism, and DCI

No matter any possible deleterious effects related to enhanced ROS and RNS formation, pure O_2_ breathing is the therapy of choice during CO intoxication, gas embolism, and DCI. While the beneficial effect of hyperoxia during CO intoxication is related to the competitive replacement of CO in heme moieties, the salutary role of O_2_ during DCI and/or gas embolism is due to the so-called oxygen window effect.

Recent reports show that approx. 1 ‰ of patients admitted to emergency departments present with occult CO-intoxications [91]. CO has a several-fold higher affinity to heme moieties than O_2_, and thus it reduces the blood O_2_ transport capacity by preventing hemoglobin (Hb) O_2_ saturation. This effect on tissue O_2_ transport is further aggravated by the leftward-shift of the Hb-O_2_-dissociation curve, which impairs O_2_ release from oxy-hemoglobin [92]. Nevertheless, CO toxicity is mainly due to the blockade of complex IV of the mitochondrial respiratory chain (i.e., cytochrome c oxidase) [93]. Ultimately, this inhibition of mitochondrial respiration will result in oxidative and nitrosative stress [94], which also explains that pure O_2_ breathing is the therapy of choice in patients with CO intoxication: albeit at first glance paradoxical, increasing the PO_2_ in fact reduces rather than further increases ROS and RNS formation during CO intoxication [94], because high O_2_ concentrations will restore normal electron transport within the respiratory chain and thereby decrease radical production. The half-life of CO elimination is inversely related to the arterial PO_2_ [95], and therefore, intuitively, HBO therapy is indicated in patients with CO-intoxication. However, the results of the available RCT are equivocal [96–98], and a recent meta-analysis concluded that normobaric hyperoxia is as efficient as HBO, in part as a result of the CO elimination achieved with normobaric pure O_2_ breathing during patient transport to an HBO chamber [99].

**Fig. 3:**
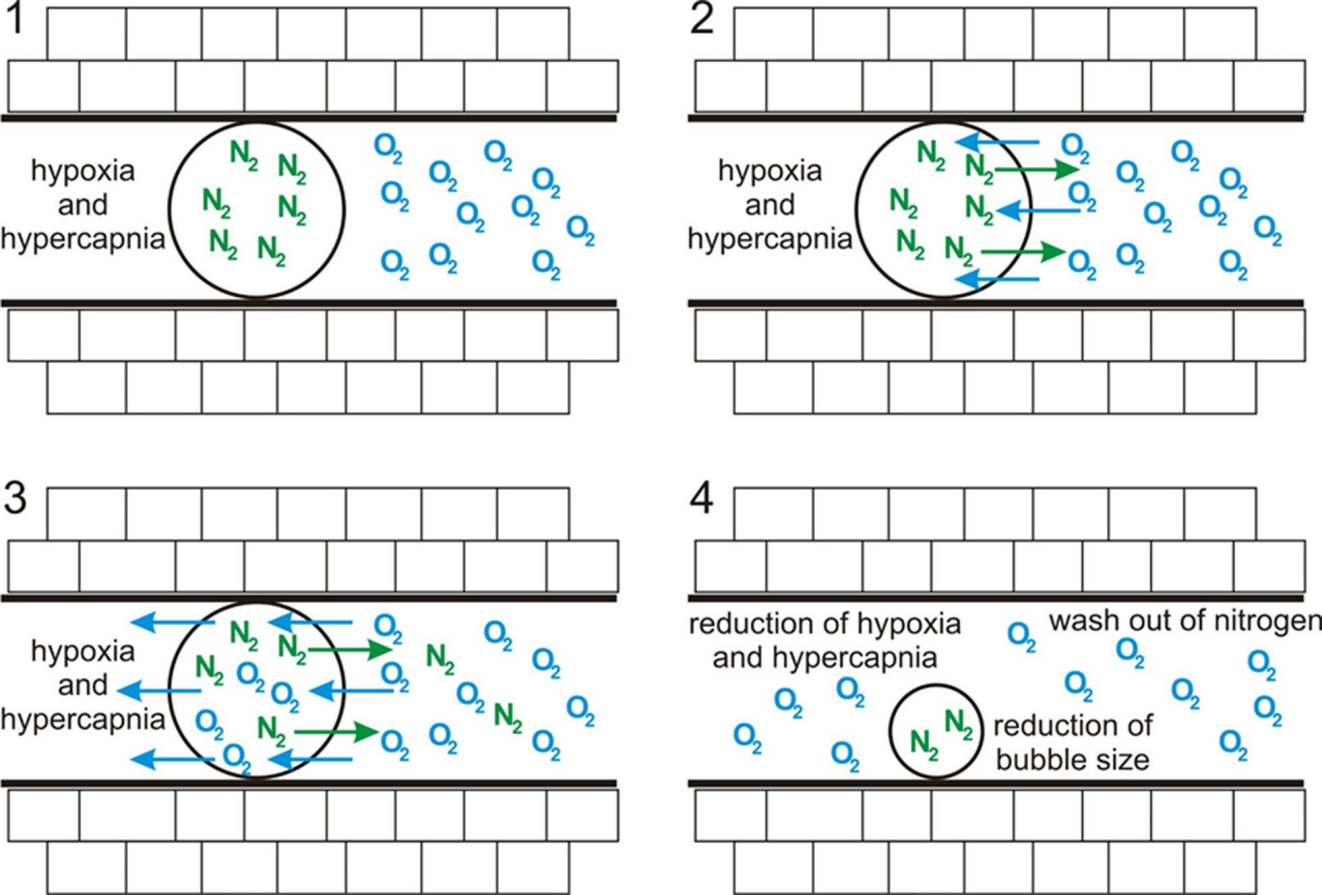
Effect of increased oxygen partial pressure on bubble size. After creation of a concentration gradient (**1**), oxygen starts to diffuse into the bubble, simultaneously nitrogen diffuses out of the bubble (**2**). Thereby, oxygen molecules are now capable of passing the bubble with concomitant reduction of nitrogen concentration (**3**). Finally, the bubble size decreases significantly (**4**). Adapted from Muth et al. [103] with kind permission from Springer Science and Business Media

By definition gas embolism is the—mostly iatrogenic—entry of gas bubbles into the vascular system in general [100], whereas decompression injury (DCI) comprises medical disorders resulting from a decrease in ambient pressure (i.e., decompression) that results in intra- or extra-vascular bubble formation due to excess (i.e., supersaturation) inert gas (in most cases N_2_) tensions [101]. However, DCI can also cause arterial gas embolism due to introduction of alveolar gas emboli via cardiac shunts and/or pulmonary vessels, but more frequently presents as decompression sickness (DCS), which is caused by excess supersaturation during and after decompression [102]. Treatment is breathing 100 % O_2_, and, as far as DCI is concerned, in combination with recompression, i.e., HBO [103]. In addition to its ability to improve tissue oxygenation and attenuate inflammation, pure O_2_ breathing is therapy of choice because it maximizes the inert gas gradient from the tissues to the alveolar gas and thereby accelerates inert gas washout [102]. Moreover, it will enhance bubble resolution due to the increased inert gas diffusion gradient (i.e, the oxygen window) (Fig. 3 [103]).

### Acute coronary syndrome

In 1940, supplemental O_2_ breathing was described as “…as an efficient method of relieving the intense pain which may accompany acute coronary thrombosis and as an important therapeutic adjunct in the symptomatic control of severe angina pectoris…” [104], and subsequently became a cornerstone of the management of the Acute Coronary Syndrome. However, due to the above-mentioned coronary vasoconstriction [83], which was also more recently demonstrated to be due to NO quenching [105] associated with oxidative and nitrosative stress [73], this approach has been questioned [106, 107], despite data from patients with acute decompensated heart failure showing no effect of the arterial PO_2_ on all-cause mortality [108]. Therefore, the latest guidelines of the European Resuscitation Council on the initial management of acute coronary syndromes recommend that an “…O_2_ saturation of 94–98 %, or 88–92 % if the patient is at risk of hypercapnic respiratory failure…” should be achieved, in other words, “…supplementary O_2_ should be given only to those patients with hypoxaemia, breathlessness or pulmonary congestion…” [109]. Until most recently, the evidence for these guidelines was surprisingly scarce, because over four decades only four clinical trials enrolling a total of just 447 patients were published [110–113]. Moreover, the results of these trials were far from being conclusive: In 17 patients with anterior transmural myocardial infarction (MI), Madias et al. reported reduced ischemic injury as assessed by precordial ST-mapping during 48–80 min of breathing 15 L/min O_2_; however, this study did not include any control group [110]. In 157 patients with confirmed MI, Rawles et al. found no difference in the incidence of arrhythmias and use of analgesics after 6 L/min O_2_ over 24 h vs. air breathing; however, mortality in the O_2_-group tended to be higher without reaching statistical significance (3.9 vs. 11.3 %, p = 0.08) [111]. More recently, in a total of 137 patients using two different protocols for supplemental O_2_, Ukholkina et al. demonstrated that a FiO_2_ of 0.3–0.4 until 3 h after interventional myocardial revascularization decreased the number of early post-intervention arrhythmia, which was associated with lower peak values of myocardial creatine kinase activity, and, ultimately, smaller relative area of ischemic damage. However, 37 % of the patients had baseline O_2_ saturations <94 %, i.e., below the threshold recommended for initiating supplemental O_2_ administration. Moreover, for reasons unexplained, time to revascularization was longer in the O_2_-group [112]. Finally, Ranchord et al. found “…no evidence of benefit or harm…” from high-concentration (6 L/min O_2_ over 6 h) vs. titrated O_2_ therapy (to achieve O_2_ saturations of 93–96 %) in 136 patients with initially uncomplicated ST-elevation myocardial infarction [113]. Therefore, as highlighted in recent reviews [106, 107], there is urgent need for large clinical trials assessing whether or not O_2_ therapy should be used for the management of acute coronary syndrome, and the results of the DETO2X-AMI (NCT01787110) (see Table 1) trial is to answer this question. The most recently completed AVOID (NCT01272713) (see Table 1) trial has partly answered this need: in non-hypoxaemic (transcutaneous hemoglobin O_2_ saturation >94 %) patients with ST-elevation myocardial infarction (n = 441), high flow face mask O_2_ (8 L/min) up to 4 h after percutaneous coronary intervention increased myocardial injury, recurrent infarction, major cardiac arrhythmia, and late (6 months) myocardial infarct size. Mortality at hospital discharge did not significantly differ (p = 0.11), but interestingly, was 2.5 fold higher in the normoxia group. At 6 months, however, both overall (hyperoxia: 3.8, normoxia: 5.9 %; p = 0.32) and cardiac (hyperoxia: 2.9, normoxia 4.1 %) mortality were comparable [114].

### Traumatic and ischemic brain injury

From a pathophysiological point of view, any hyperoxia-induced vasoconstriction could theoretically represent an interesting approach in the management of brain injury, inasmuch as it would allow reducing intracranial pressure (ICP) and thereby improving cerebral perfusion pressure without impairment of O_2_ supply. Clearly, HBO (60 min of mechanical ventilation with pure O_2_ at 1.5 atmospheres of ambient pressure was shown to efficiently decrease ICP in patients with traumatic brain injury (TBI) [115]. Combining HBO with subsequent normobaric hyperoxia even improved long-term outcome: at 6 months mortality was reduced (9 out of 22 vs. 3 out of 20 patients, p = 0.048), and overall neurological outcome was more favorable as evaluated with the sliding dichotomized Glasgow Outcome Score (8 out of 21 vs. 14 out of 19 patients, p = 0.024) [116]. However, normobaric hyperoxia alone yielded equivocal results with respect to tissue oxygenation and metabolism as assessed by microdialysis [117–122], which was referred to a lacking effect on brain tissue oxygenation in hypo-perfused regions [120] and/or a possibly enhanced hyperoxia-related excitotoxicity [122]. Albeit there is some data available using magnetic resonance imaging (MRI), suggesting that hyperoxia may have a beneficial effect in the peri-lesional penumbra [123], the role of hyperoxia in TBI is still controversially discussed because of the equivocal outcome data [124]: while a uni-variate analysis found a significant association between hyperoxemia (arterial PO_2_ >100 mmHg) and a decreased risk of 6-month mortality in a retrospective analysis of 1116 patients, the corresponding multi-variate logistic regression adjusted for illness severity did not show any significant relationship [125]. However, Davis and co-workers showed in a large retrospective cohort analysis including 3420 patients that both hypoxemia (PaO_2_ < 110 mmHg) and extreme hyperoxemia (PaO_2_ > 487 mmHg) were associated with increased mortality and unfavorable outcome among TBI patients [126]. Moreover, two other retrospective studies analyzing a total of 1759 patients using multi-variate approaches showed that hyperoxemia defined as arterial PO_2_ >200 or >300 mmHg, respectively, was independently associated with *increased* mortality [127, 128]. These data are in contrast to another retrospective study, reporting that oxygen partial pressures between 250 and 486 mm Hg were associated with improved all-cause survival in patients with severe TBI [129]. So far, the answer to the question of the use of hyperoxia in TBI is still pending: the BRAINOXY study (NCT01201291), which was to answer this question, was terminated due to slow recruitment.

The currently available data on hyperoxia (with consecutive hyperoxemia) during ischemic brain injury, i.e., stroke and/or intracranial bleeding, is less conflicting: Albeit there is compelling experimental evidence (for review, see [130]) and some encouraging pilot data in patients [131, 132], evidence from large trials suggests that hyperox(em)ia is deleterious. A prospective, single-center observational study in 252 patients showed that hyperoxemia (as defined as a PaO_2_ >173 mmHg) was associated with delayed cerebral ischemia and, consequently, poor neurological outcome [133]. In addition, a more recent retrospective analysis of 2894 mechanically ventilated patients with ischemic stroke, subarachnoid or intracerebral hemorrhage demonstrated that more pronounced hyperoxemia (arterial PO_2_ >300 mmHg) significantly increased in-hospital mortality at day 28 [134]. In contrast, a retrospective analysis of 2,643 adults, ventilated for ischemic stroke in ICUs in Australia and New Zealand, showed no apparent relationship between mortality and PaO_2_ levels during the first 24 h in ICU [135]. Finally, the Normobaric Oxygen Therapy in Acute Ischemic Stroke Trial (NCT00414726), which was to study the effects of high-flow O_2_ (30–45 L/min for 8 h via facemask) was terminated prematurely after enrolment of 85 of 240 patients due to imbalance in deaths favoring the control arm (hyperoxia: 17 out of 43 patients, room air: 7 out of 42 patients, p = 0.03). The question, however, whether hyperoxia is definitely deleterious, remained unanswered: deaths were not attributed to treatment by the blinded external medical monitor. No matter the impact of high-flow supplemental O_2_, even low-dose O_2_ administration (2 L/min either continuously over 72 h or over-night only) only targeted to compensate for mild, in particular nocturnal, hypoxemia (Stroke Oxygen Study, *SO*_*2*_*S*; ISRCTN52416964) did not improve outcome after ischemic stroke: despite promising pilot data in 289 patients at 1 week and 6 months [136, 137], the complete, full-scale study in 8003 patients did not show any difference in morbidity (disability at day 90 as assessed by the modified Rankin Scale) or mortality (data presented at the XXIII European Stroke Conference, Nice, May 7, 2014).

### Cardiac arrest

The pronounced vasoconstrictor effect in the cerebral circulation together with the potential to aggravate oxidative stress during ischemia/reperfusion have prompted investigations on the association between hyperox(em)ia and outcome after cardiopulmonary resuscitation. So far only retrospective analyses are available, except for one randomized controlled single centre trial including 28 patients in total, the results being again fairly equivocal: a multicenter cohort study on 6326 patients concluded that hyperoxemia defined as PaO_2_ >300 mmHg was associated with higher mortality than normoxemia and even hypoxemia defined as PaO_2_ <60 mmHg) [138]. A secondary analysis of 4459 patients of this study even yielded a direct linear relationship between PaO_2_ increments and increased risk of mortality, a PaO_2_ increment of 100 mmHg being associated with a 24 % higher odds ratio for unfavorable outcome [139]. Another retrospective analysis of 12,108 patients found no association between PaO_2_ deciles or hyperoxemia defined as PaO_2_ >309 mmHg and mortality adjusted for illness severity [140]. Other authors analyzing smaller data bases confirmed this latter finding [141, 142]. Clearly, different temperature management (lowest temperature: median 34.9 °C in [140] vs. mean 36 °C in [138]; 33 vs. 6 % of patients <34 °C) in the various countries may have contributed to these divergent findings, albeit a single-center, retrospective analysis of 170 patients treated with therapeutic hypothermia (12–24 h at 32–34 °C core temperature) showed that mortality and poor neurological outcome were more frequent in patients with higher maximum PaO_2_ values (median 254 vs. 198 mmHg) during the first 24 h after cardiac arrest [143]. This is in line with another retrospective analysis in 213 patients after cardiac arrest, treated with therapeutic hypothermia, demonstrating a U-shaped independent association between the mean PaO_2_ and poor neurologic outcome at hospital discharge [144]. The sole randomized controlled trial, comparing 14 patients in each group ventilated with either 30 or 100 % oxygen for 1 h after return of spontaneous circulation (ROSC), showed increased levels of neuron specific enolase in the hyperoxic group at 24 h post cardiac arrest [145]. Unfortunately, this study was not powered to analyze outcome parameters. Finally, other smaller studies focusing on the role of arterial PCO_2_ did not yield any deleterious effect of hyperoxemia per se on neurological outcome [146–148]. Consequently, a recent meta-analysis concluded that hyperoxemia (PaO_2_ >300 mmHg) “…appears to be correlated with increased in-hospital mortality…”, which, however, “…should be interpreted cautiously because of the significant heterogeneity…of studies analyzed…” [149]. Lately, two more interesting retrospective cohort analyses reported that severe hyperoxia was associated with decreased survival as well as decreased survival and worse neurological outcome, respectively [65, 150]. Nelskylä et al. offer an interesting explanation for the vast majority of retrospective studies being in favor of normoxia: In their retrospective analysis of 119 out of hospital cardiac arrest patients, hyperoxia occurred more frequently in association with out-of-hospital cardiac arrest, longer times to ROSC, and delays to ICU admission, i.e., the patients with the worst prognosis per se [142]. In addition, safe titration of oxygen therapy to achieve a SpO_2_ of 90–94 % after out-of-hospital cardiac arrest might not be feasible, at least in the pre-hospital period [151]. Taken together, all these studies demonstrate the urgent need for data from prospective, randomized controlled trials, and the ongoing REOX trial (NCT01881243) will at least help to answer this demand.

### Peri-operative hyperoxia

The use of intra-operative (and, in a broader sense, peri-operative) hyperoxia to prevent surgical site infection does not directly refer to the treatment of circulatory shock and medical emergencies, but patho-physiological effects of hyperoxemia also assume importance in this context. The antimicrobial properties of oxygen are due to the bactericidal properties associated with increased ROS production, and were already recognized in the 1980s (“oxygen as an antibiotic” [152]), subsequently prompting several clinical studies which have so far enrolled more than 5000 patients. Recent meta-analyses of these studies concluded that high inspired O_2_ concentrations values (FiO_2_ = 0.8 vs. 0.3 as the standard approach) during the peri-operative period reduced the risk of surgical site infection, both after elective and emergency surgery, without leading to major post-operative atelectasis [153, 154]. This protective effect was specifically present in non-obese patients undergoing colo-rectal surgery, one possible component being a better patency of anastomoses [155]. The molecular mechanisms of hyperoxia-related reduction in surgical site infection remain unclear: hyperoxic ventilation was reported to restore the local inflammatory response to normal—rather than leading to potentially deleterious hyper-inflammation—thereby improving the antimicrobial potential of alveolar macrophages [156]. However, other authors found that ex vivo exposure to hyperoxia not only enhanced ROS formation but even decreased the capacity of endotoxin-stimulated leukocytes to release tumor necrosis factor-α [157]. It is noteworthy that despite this short-term (up to 2 weeks within surgery) benefit, high intra-operative FiO_2_ was associated with higher long-term (>2 years) post-operative mortality. This observation was nearly exclusively due to a higher mortilaty in patients that had undergone cancer surgery [158], and coincided with a significantly shorter cancer-free survival interval [159]. Therefore, and taking into account the trials showing no benefit for surgical site infection after abdominal surgery [160, 161], the most recent Cochrane analysis concluded that “…evidence is insufficient to support the routine use of a high fraction of inspired O_2_ during anesthesia and surgery….” [162].

### What is the optimal PaO_2_ for ICU survival?

So far, this question remains unanswered as well: a retrospective analysis of arterial PO_2_ measurements in 36,307 patients during the first 24 h of ICU stay demonstrated a U-shaped relationship of in-hospital mortality, the nadir of the mortality curve (as calculated from the logistic regression with the PaO_2_ incorporated using a spline function) being at values of 15–20 kPa (110–150 mmHg); mortality sharply increased both at PaO_2_ values <9 (67 mmHg) and >30 kPa (225 mmHg) [163]. Interestingly, this group of authors recently showed a similar U-shaped relation between arterial PCO_2_ and PO_2_, respectively, and hospital mortality after cardiac arrest, the highest probability of survival being associated with a PaO_2_ values of 180–200 mmHg, i.e., most likely with an FiO_2_ >0.6 in a substantial number of patients [164]. A more recent study of unadjusted odds ratios for PaO_2_ deciles in 152,680 patients confirmed the impact of hypoxemia, whereas hyperoxemia even >40 kPa (300 mmHg) had no impact on outcome [165]. Finally, a retrospective cohort study including 83,060 patients after cardiac surgery showed that there was no association between mortality and hyperoxia in the first 24 h in ICU after cardiac surgery [166]. Therefore, two recent meta analyses concluded that hyperoxia may be associated with increased mortality in patients with stroke, TBI, and post cardiac arrest and with poor hospital outcome, respectively [167, 168]. However, due to heterogeneity of the included studies, the authors state that more evidence is needed to provide optimal oxygen targets for critical care physicians. The results of the OXYGEN-ICU (NCT01319643) trial (see Table 1) will certainly contribute to the answer of this question. Interestingly, most ICU clinicians acknowledge the potential adverse effects of prolonged exposure to hyperoxia, however, in actual clinical practice, a large proportion of their patients was exposed to higher arterial oxygen levels than self-reported target ranges [169].

## Conclusion

Hyperoxia (i.e., ventilation with a FiO_2_ = 1.0) is a cornerstone of the acute management of circulatory shock, a concept which is based on compelling experimental evidence that compensating the imbalance between O_2_ supply and requirements (i.e., the oxygen dept) is crucial for survival, at least after trauma [170, 171]. On the other hand oxygen toxicity due to the increased formation of ROS limits its use, because it may cause serious deleterious side effects, especially in conditions of ischemia/reperfusion. While these effects are particularly pronounced during long-term administration, i.e., beyond 12–24 h, several retrospective studies suggest that even hyperoxemia of shorter duration is also associated with increased mortality and morbidity. In fact, albeit the clinical evidence from prospective studies is surprisingly scarce, a recent meta-analysis suggests that hyperoxia is associated with increased mortality at least in patients after cardiac arrest, stroke and TBI [172]. Most of these data, however, originate from heterogenous, observational studies with inconsistent results, and therefore, there is a need for the results from the large scale, randomized, controlled clinical trials on the use of hyperoxia, which can be anticipated within the next 2–3 years. Consequently, until then, “…conservative…” O_2_ therapy [140] represents the treatment of choice to avoid exposure to both hypoxemia and excess hyperoxemia.

## Abbreviations

ARDS: Acute Respiratory Distress Syndrome
ATP: adenosine triphosphate
CO: carbon monoxide
DCI: decompression injury
DCS: Decompression sickness
ECG: electrocardiogram
FiO_2_: fraction of inspired oxygen
Hb: hemoglobin
HBO: hyperbaric oxygenation
ICP: intracranial pressure
ICU: intensive care unit
MI: myocardial infarction
MRI: magnetic resonance imaging
N_2_: nitrogen
NO: nitric oxide
O_2_: oxygen
PaO_2_: arterial partial pressure of oxygen
PCO_2_: partial pressure of carbon dioxide
PEEP: Positive end-expiratory pressure
PO_2_: partial pressure of oxygen
RCT: randomized controlled trial
RNS: reactive nitrogen species
ROS: reactive oxygen species
ROSC: return of spontaneous circulation
SIRS: systemic inflammatory response syndrome
SpO_2_: peripheral hemoglobin oxygen saturation
TBI: traumatic brain injury
V_A_/Q: ventilation/perfusion ratio
VILI: ventilator-induced lung injury
